# Epitope-based antibody development against metalloproteinases and phospholipases A_2_ from *Deinagkistrodon acutus* venom

**DOI:** 10.1590/1678-9199-JVATITD-2024-0060

**Published:** 2025-05-09

**Authors:** Haiting Zhu, Yuexin Pan, Zhiyuan Tai, Mingqian Wang, Xia Liu, Xiaodong Yu, Qiyi He

**Affiliations:** 1 Engineering Research Center of Active Substance and Biotechnology, Ministry of Education, College of Life Science, Chongqing Normal University, Chongqing, China.

**Keywords:** Snakebite envenoming, Immunization, Recombinant proteins, Antigenic epitopes, Therapeutic antibodies

## Abstract

**Background:**

*Deinagkistrodon acutus*, or the hundred-pace snake, poses severe health risks due to its venom. Envenomation by this snake leads to complications such as hemorrhage, edema, and coagulopathy. Traditional antivenoms are limited by venom variability and often contain non-neutralizing antibodies, highlighting the need for more precise and effective immunogens.

**Methods:**

This study utilized epitope-based antibody technology to develop a targeted sera against venom metalloproteinases (MPs) and phospholipases A_2_ (PLA_2_s). Twelve antigenic epitopes were identified via bioinformatics, leading to the design of a composite antigen peptide, EpiMPLA. It was engineered to be expressed via two expression systems, resulting in the recombinant immunogens, ProMPLA and p2AMPLA.

**Results:**

Immunization with ProMPLA and p2AMPLA produced robust antibody responses in mice, effectively inhibiting MPs and PLA_2_s. *In vitro* assays demonstrated that sera from immunized mice reduced the activity of these venom enzymes, minimized venom-induced hemorrhage and edema, and restored blood coagulation. At a venom dose of 2×LD_50_, all mice in the control group died, while survival rates were 90% for anti-ProMPLA and 70% for anti-p2AMPLA.

**Conclusion:**

The EpiMPLA epitope represents a promising candidate for generating neutralizing antibodies against *D. acutus*venom, demonstrating its potential to address critical gaps in current antivenom therapy. These findings not only validate the feasibility of epitope-based antivenom development but also pave the way for further research to optimize this strategy.

## Background

*Deinagkistrodon acutus*, commonly known as the “hundred-pace snake” or “five-pace viper,” belongs to the Viperidae family and is predominantly distributed across Taiwan, mainland China (spanning the provinces of Zhejiang, Fujian, Guizhou, southeastern Sichuan, Hubei, and Hunan), and northern Vietnam (including Yen Bai and Ha Giang) [1]. Envenomation by *D. acutus* can lead to severe clinical manifestations, including acute hemorrhage, edema, coagulopathy, hemolysis, and tissue necrosis. In particularly severe cases, such envenomation may result in ischemic shock and death, highlighting the critical need for effective therapeutic interventions [2-4].

The acute toxicity of *D. acutus* venom is primarily attributed to the presence of hemorrhagic toxins and platelet aggregation inhibitors, including metalloproteinases (MPs) and phospholipase A_2_ enzymes (PLA_2_s) [5]. Metalloproteinases, which are part of the zinc-dependent metalloproteinase family, play a pivotal role in the pathophysiology of envenomation. They degrade components of the capillary basement membrane, such as collagen and laminin, increasing vascular permeability and resulting in localized hemorrhage and edema [6-8]. Conversely, PLA_2_s hydrolyze phospholipids in cell membranes, destabilizing them and causing the release of lysophospholipids and fatty acids. This can lead to membrane rupture, hemolysis, muscle damage, and localized inflammation [9]. Furthermore, PLA_2_s inhibit ADP-induced platelet aggregation and impede acetylcholine release from nerve terminals, contributing to both local and systemic coagulation disorders [10-12]. The synergistic effects of these toxins exacerbate hemorrhage and muscle damage, leading to the release of myoglobin and other cellular components into circulation. When filtered through the kidneys, these components may precipitate acute tubular necrosis and renal failure.

Given the severity of envenomation, the development of specific antibodies targeting these toxins is crucial for effective treatment. In Taiwan and mainland China, monovalent antivenoms are employed to treat snakebites [13]. These antivenoms are produced by immunizing horses or sheep with snake venom. Although these antibodies can recognize various venom components, they also contain significant amounts of non-neutralizing immunoglobulins, necessitating larger doses for effective treatment [14]. Furthermore, the composition of the venom used as antigen varies according to factors such as geography, age, and sex, which contributes to significant differences in antivenom potency across different batches [15-17]. While mixing venoms from various geographical locations, ages, and sexes may mitigate some of these issues, it does not fully resolve the problem of non-specific antibodies in antivenoms [18]. Thus, there is an urgent need for more effective immunogens to enhance the quality and efficacy of antivenoms.

In this context, epitope-based antibody technology emerges as a promising biotechnological approach with wide applications in vaccine development, diagnostic reagent production, and immunotherapy [19]. This technology enables precise recognition and binding to target antigen epitopes, improving the specificity and efficacy of sera while minimizing non-specific binding and associated side effects [20]. Current research on epitope-based antibody production primarily focuses on two methodologies: recombinant protein immunization and gene immunization. In recombinant protein immunization, bacterial expression systems, such as *Escherichia coli*, are utilized to produce epitope peptides as recombinant proteins, effectively stimulating rapid immune responses. For instance, Figueiredo et al. [21] successfully expressed and purified a recombinant protein (rCpLi) containing three epitopes derived from *Loxosceles intermedia* spider venom. The resulting sera exhibited reactivity comparable to that generated from crude venom immunization and effectively bind toxins and inhibit skin necrosis activity. Similarly, Ramos et al. [22] expressed and purified a recombinant protein containing potential B-cell epitopes from *Micrurus corallinus* three-finger toxins and PLA_2_ toxin linear epitopes, significantly enhancing immunity in mice and achieving a 60% survival rate following coral snake envenomation. Gene immunization, conversely, involves the insertion of antigen-encoding genes into plasmids or viral vectors, enabling host cells to express the corresponding antigens and induce both humoral and cellular immune responses. For example, Meas et al. [23] developed a bivalent vaccine against porcine circovirus type 2 (PCV2) using epitope prediction technology, which successfully induced neutralizing antibodies that inhibited PCV2 cell entry. In another study, Menzies et al. [24] used BepiPred to predict the epitopes of phospholipase and three-finger toxins, and the epitope design was based on the conservation and accessibility of the toxin sequences. Subsequent studies found that virus-like particles presenting snake venom epitopes induced antibody responses in mice, successfully identifying geographically and taxonomically different ranges of snake venom.

The application of epitope-based antibody technology in antivenom production offers several key advantages. First, high-affinity antigenic regions of venom components can be rapidly identified based on protein sequences, facilitating the production of antivenoms that address batch variability arising from differences in venom composition. Second, integrating this technology with nanotechnology enhances epitope targeting and immune activation. For example, nanocarriers improve stability, bioavailability, and targeted delivery of epitope-based antibodies, while nanovaccines optimize epitope presentation and immune activation, boosting antivenom efficacy. Lastly, it enables the incorporation of key antigenic regions from multiple toxins, potentially aiding in the development of polyvalent antivenoms targeting various snake species [25-28].

Despite these advancements, research on the antigenic epitopes of *D. acutus* venom remains limited. For instance, Cao et al. [29] utilized DNAStar and IEDB to predict the linear B-cell epitopes of major toxins from *D. acutus*, resulting in the development of a sera that reduced venom-induced hemorrhagic activity. However, the metalloproteinase sequences used in their predictions were underrepresented, covering only one-third of the total sequences available in the UniProt database (e.g., only four sequences were used, including repeated entries such as AY566610, GQ245980, and AJ223283). This limited representation does not reflect the actual diversity of metalloproteinases in venom. Furthermore, their study focused solely on antigenicity in epitope prediction, overlooking the functional roles of key toxic fragments, such as zinc ion active sites, integrin domains, and integrin-like domains, which are critical for the structural and functional integrity of metalloproteinases [30]. 

In this study, we utilized the Immune Epitope Database (IEDB) to predict and analyze the antigenic epitopes of MPs and PLA_2_s derived from *D. acutus* venom. The IEDB provides a wealth of information on epitopes associated with infectious diseases, allergic responses, and antigen interactions, including antibody data for humans, mice, non-human primates, and other species [31]. To generate immune sera, we employed both recombinant protein immunization and gene immunization approaches. The immunoreactivity of the resulting sera against *D. acutus* venom was evaluated using enzyme-linked immunosorbent assay (ELISA) and Western blot (WB) analysis. *In vitro* assays were conducted to evaluate the inhibitory effects of these sera on the enzymatic activities of MPs and PLA_2_s. Additionally, their efficacy in reducing venom-induced hemorrhage, hemolysis, and edema, as well as their ability to mitigate venom-induced coagulation disturbances *in vivo*, was investigated. Finally, the protective effects of the sera were evaluated in a mouse model.

## Methods

### Materials and reagents

*D. acutus* snake venom protein was provided by the Biotechnological Engineering Research Center of Active Substances, Ministry of Education. DH5α chemically competent cells and BL21 Star (DE3) chemically competent cells, as well as the DNA Gel Extraction Kit, were purchased from Tsingke Biotech Co., Ltd. (Beijing, China). The SDS-PAGE Gel Preparation Kit, BCA Protein Assay Kit, Seamless Cloning Kit, Freund's Complete Adjuvant (FCA), Freund's Incomplete Adjuvant (FIA), HRP-labeled Goat Anti-Mouse IgG (H+L), TMB Substrate Solution, and BeyoECL Plus were all sourced from Beyotime Biotechnology Co., Ltd. (Shanghai, China). Rehydragel® LV Alum Adjuvant was acquired from Bioesn Biotechnology Co., Ltd. (Shanghai, China). The azocasein was purchased from Sigma-A50rich (Dublin, Ireland), and the coagulation test kit was obtained from Wuhan Zhongtai Biotech Co., Ltd. (Wuhan, China). All other reagents used were of analytical grade.

### Antigen epitope prediction and synthesis

The amino acid sequences of *D. acutus* MPs and PLA_2_s were retrieved and downloaded from the UniProt database (https://www.uniprot.org/ ). After removing the signal peptide and propeptide sequences, the mature peptide regions were subjected to linear B-cell epitope prediction using the IEDB-Bepipred 2.0 online server (http://tools.iedb.org/bcell/) [32-34]. The toxin amino acid sequences were then aligned and analyzed using the Alignment module of MEGA software (https://mega.co.nz/). Antigen epitopes were selected based on high antigenicity, sequence homology, and conserved motifs. These selected epitope fragments were linked together using KK linkers, and the resulting sequence was reverse translated into a nucleotide sequence. Codon optimization was performed, and restriction sites for *Bam*H I (5' end) and *Hin*d III (3' end) were added. The final nucleotide sequence was synthesized by Beijing Tsingke Biotech.

### Prokaryotic expression and purification of antigen epitopes

The synthesized nucleotide sequence encoding the antigen epitopes was ligated into the pET28a (+) plasmid to construct a recombinant prokaryotic expression plasmid. The plasmid was then transformed into BL21 Star (DE3) chemically competent cells, and protein expression was induced using isopropyl-beta-D-thiogalactopyranoside (IPTG). After induction, the bacterial culture was centrifuged at 5,000 rpm for 20 min, and the pellet was collected. For each liter of bacterial culture, 80 mL of denaturing lysis buffer was added to resuspend the bacterial pellet. The suspension was subjected to three freeze-thaw cycles in liquid nitrogen, followed by sonication on ice for 20 min (300 W, JY92-IIN, Ningbo Scientz Biotechnology, China). The bacterial lysate was then centrifuged at 10,000 *g* for 20 min at 4°C, and the supernatant containing the target protein was collected.

The expressed antigen epitope protein was purified using BeyoGold™ His-tag Purification Resin (Beyotime Biotechnology, China), and the protein concentration was determined using the BCA Protein Assay Kit (Beyotime Biotechnology, China), following the manufacturer's instructions. The purity and molecular weight of the protein were analyzed by 12% SDS-PAGE gel electrophoresis (Bio-Rad, USA). After desalting via ultrafiltration, the protein was freeze-dried using a freeze dryer (SCIENTZ-18N, Ningbo Scientz Biotechnology, China) and stored at -20°C until further use.

### Construction and extraction of eukaryotic expression plasmid

Following the protocol provided in the Seamless Cloning Kit, the synthesized nucleotide sequence encoding the antigen epitopes was seamlessly inserted into the Ig*κ* leader sequence of the pSecTag2A plasmid, resulting in the creation of a recombinant eukaryotic expression plasmid. This plasmid was then transformed into DH5α chemically competent cells. Positive clones were screened on LB solid medium containing 100 μg/mL of ampicillin. After colony selection, the plasmids were extracted from cultured positive clones, and the nucleotide sequence was verified through sequencing to confirm the correct insertion.

Subsequently, clones with the correct sequence were selected for large-scale culture, and plasmids were extracted using standard plasmid purification methods. The concentration of the extracted plasmids was then measured with a Nano-100 Micro-Spectrophotometer (Beijing YPH Biotechnology, China), followed by freeze-drying using the SCIENTZ-18N freeze dryer. The plasmids were stored at -80°C until further use.

### Mouse immunization and serum collection

Healthy female Kunming (KM) mice, aged 6-8 weeks and weighing 19 ± 1.0 g, were obtained from Chongqing Ensiweier Laboratory Animal Sales Co., Ltd. (Chongqing, China). The mice were maintained under standard husbandry conditions, including a controlled environment with 50% to 70% relative humidity, an ambient temperature of 25 to 26 ℃, and a 12-hour light/dark cycle. They were provided with *ad libitum* access to standard feed and sterile water. All experiments were conducted in strict compliance with the National Institutes of Health's Guide for the Care and Use of Laboratory Animals and received approval from the Ethics Review Committee at the College of Life Sciences, Chongqing Normal University (Code: #2024008).

Immunization protocols were carried out following the method described by Figueiredo et al. [21] and Meas et al. [23], with slight modifications for recombinant protein and plasmid administration. A total of 48 female Kunming (KM) mice were randomly assigned to four groups (A, B, C, and D), with 12 mice in each group. For group A, the recombinant protein was diluted in phosphate-buffered saline (PBS, pH 7.2) to a final concentration of 2 μg/μL. The diluted protein was then emulsified with an equal volume of adjuvant to create an oil-in-water emulsion. A total of 100 μL of this emulsion was injected subcutaneously into four sites per mouse: 20 μL/part into the back, bilateral axillae, and groin. Group B served as the negative control for group A and received PBS buffer mixed with adjuvant in the same manner. For group C, the recombinant plasmid was similarly diluted in PBS buffer to a concentration of 2 μg/μL and mixed with an equal volume of adjuvant to form a gel. A total of 100 μL of this gel mixture was injected intramuscularly into the limbs, with 25 μL/part administered into each of the four limbs. Group D, the negative control for group C, received the empty pSecTag2A plasmid mixed with adjuvant, injected in the same way.

The immunization regimen consisted of three injections and administered at two-week intervals. For groups A and B, the first immunization used Freund's Complete Adjuvant (FCA), followed by Freund's Incomplete Adjuvant (FIA) for the subsequent two injections. In groups C and D, aluminum hydroxide adjuvant was used for all immunizations. Seven days after each immunization, blood samples (approximately 150 µL per mouse) were collected via retro-orbital sinus puncture under anesthesia to minimize animal discomfort. The collected blood was centrifuged at 3,000 g for 10 min, and the serum was separated and stored at -80°C for future analysis. 

### ELISA analysis

An indirect ELISA was conducted to evaluate the production of antibodies in mice throughout the immunization period, following the method described by Liu et al. [35], with minor modifications. Briefly, 96-well microplates were coated with 100 μL of *D. acutus* venom protein (100 ng/well), diluted in 0.1 M carbonate buffer (pH 9.6), and incubated overnight at 4°C. After the coating step, the wells were washed three times with PBS buffer containing 0.05% Tween-20 (PBST) to remove any unbound protein. The wells were then blocked by adding 200 μL of PBST containing 3% BSA to prevent nonspecific binding. The plates were incubated at 37°C for 2 h. Following another round of three washes with PBST, 100 μL of diluted mouse serum, prepared in PBST with 1% BSA, was added to each well and incubated at 37°C for 2 h. After serum incubation, the plates were washed three more times, and 200 μL of HRP-conjugated goat anti-mouse IgG secondary antibody (diluted 1:250, Beyotime Biotechnology, China), prepared in PBST with 1% BSA was added. The plates were then incubated at 37°C for 1 h.

Following this, the plates were washed three times, and 100 μL of TMB substrate solution (Beyotime Biotechnology, China) was added to each well. The color development reaction was allowed to proceed in the dark at room temperature for 20 min. The reaction was stopped by adding 100 μL of 2 M H₂SO₄ to each well, and the optical density (OD) at 450 nm was immediately measured using a SP-Max 2300A2 microplate reader (Shanghai Shanpu Biotechnology, China). All experiments were conducted in triplicate, with the results presented as the mean ± standard deviation (SD). A sample was considered positive if the A_450_ value from the experimental group exceeded 2.1 times that of the negative control group.

### Western blot analysis

Western blot analysis was conducted to evaluate the antibody responses elicited against the venom proteins of *D. acutus* [36]. Initially, 30 μg of crude venom was resolved by 12% SDS-PAGE under reducing conditions. Following electrophoresis, the proteins were transferred to a polyvinylidene fluoride (PVDF) membrane. To minimize non-specific binding, the membrane was blocked using TBST buffer containing 5% skim milk for 1 h at room temperature with gentle agitation. Subsequently, the membrane was incubated with mouse sera from each experimental group, diluted 1:2500 (v/v), for 2 h at room temperature. After three washes with TBST buffer to eliminate unbound antibodies, the membrane was incubated with a horseradish peroxidase (HRP)-conjugated goat anti-mouse IgG secondary antibody, diluted 1:1000 (v/v), for 1 h. The specific interactions between venom proteins and antibodies were visualized using BeyoECL Plus chemiluminescent substrate (Beyotime Biotechnology, China). The chemiluminescent signals were captured using an Alliance Q9 chemiluminescence imaging system (Uvitec Ltd, UK). Additionally, protein bands were stained with BeyoBlue™ Coomassie Blue Super-Fast Staining Solution (Beyotime Biotechnology, China) for 10 min at room temperature, followed by destaining to enhance band visibility. The stained bands were then examined under a gel documentation system to confirm successful protein separation and antibody binding.

### Inhibition of *D. acutus* venom metalloproteinase hydrolytic activity

The inhibitory effect of serum on the hydrolytic activity of *D. acutus* snake venom metalloproteinase (SVMP) was assessed using a modified method based on the work of Nie et al. [37], utilizing azocasein as the substrate. For the assay, a total of 5 μL of crude venom (10 μg) was mixed with serum at varying ratios of 1:1, 1:2, 1:3, and 1:4 (v/v). The total volume of each mixture was adjusted to 25 μL with PBS buffer and incubated at 37°C for 40 min. Following the incubation, the mixtures were briefly centrifuged to separate the components.

From the supernatant, 10 μL was added to 100 μL of 1% azocasein substrate, and the reaction was incubated at 37°C for an additional hour. The reaction was terminated by the addition of 300 μL of 5% trichloroacetic acid (TCA), followed by centrifugation at 8000 rpm for 10 min. Subsequently, 150 μL of the supernatant was mixed with 50 μL of 2 M sodium hydroxide (NaOH) to facilitate color development, and the absorbance was measured at 440 nm.

In the *D. acutus* venom control group, 20 μL of PBS-immunized serum was incubated with crude venom (10 μg). The SVMP hydrolysis activity in the *D. acutus* group was defined as 100%. The relative activity of SVMP in the serum-treated group was calculated as follows:

$$\text{Relative SVMP hydrolytic activity (\%) =}\frac{\;{\text{OD}}_{\text{440 (serum group)}}}{{\text{OD}}_{\text{440 (}\text{D. acutus}\text{group)}}}\text{×}\text{100\%}$$ (1)

### *Inhibition of D. acutus venom phospholipase A*_2_ hydrolytic activity

The inhibitory effect of serum on the hydrolytic activity of D. acutus snake venom phospholipase A_2_ (SVPLA_2_) was evaluated using the agar plate method adapted from Habermann et al. [38].

Initially, 0.2 g of agarose powder was dissolved in 20 mL of 0.05 M sodium acetate buffer (pH 7.5) by heating in a microwave until fully dissolved. Once the solution cooled to 50°C, 800 μL of egg yolk (prepared by mixing egg yolk with 0.85% sodium chloride solution at a 1:3 volume ratio, followed by centrifugation at 3000 rpm for 5 min to obtain the supernatant) and 400 μL of 0.01 M calcium chloride solution were added. This mixture was thoroughly mixed and then poured into a glass petri dish to solidify. After the agar solidified, holes with a diameter of 4 mm were punched into the plate.

For the assay, 5 μL of crude venom (10 μg) was mixed with serum in varying ratios of 1:1, 1:2, 1:3, and 1:4 (v/v). The total volume was adjusted to 25 μL with PBS buffer and incubated at 37°C for 40 min. After brief centrifugation, 10 μL of the supernatant was added to the wells of the agar plate, which were then incubated at 37°C for an additional 12 h to observe the results. The area of the clear zone formed around each well was measured.

In the D. acutus venom control group, 20 μL of PBS-immunized serum was incubated with crude venom (10 μg). The area of the clear zone in the D. acutus group was defined as representing 100% SVPLA_2_ hydrolytic activity. The relative activity of SVPLA_2_ in the serum-treated group was calculated as follows:

$$\text{Relative}{\text{SVPLA}}_{\text{2}}\text{s hydrolytic activity}\left(\text{\%}\right)\text{=}\frac{{\text{Area}}_{\left(\text{serum group}\right)}}{\;{{{\text{Area}}_{\text{(}\text{D. acutus}\text{group)}}}_{\;}}_{\;}}\text{×100\%}$$ (2)

### Inhibition of D. acutus venom-induced hemolysis activity

Various toxins exhibit distinct mechanisms of hemolysis. For instance, snake venom PLA_2_s hydrolyze phospholipids on red blood cell membranes, causing cell rupture [39]. MPs degrade the basement membrane and extracellular matrix, increasing vascular permeability and facilitating red blood cell rupture [7]. The inhibition effect of serum on the hemolysis induced of D. acutus snake venom was assessed using an indirect hemolysis assay, following the method described by Huang et al. [12] and Vishwanath et al. [40], with minor modifications. 

Whole blood was collected from normal mice using the eyeball exsanguination method, then subjected to centrifugation and washed multiple times with 0.9% NaCl solution to prepare a 10% red blood cell suspension. Phosphatidylcholine was suspended at a concentration of 100 mg in 100 mL of 0.01 M Tris-HCl buffer (pH 8.0), containing 0.01 M CaCl_2_ and 0.15 M NaCl. Subsequently, 1 mL of the 10% red blood cell suspension was added to 9 mL of the phosphatidylcholine suspension to create a red blood cell/phosphatidylcholine mixture.

For the assay, 5 μL of crude venom (10 μg) was mixed with serum in varying ratios of 1:1, 1:2, 1:3, and 1:4 (v/v). The total volume of each mixture was adjusted to 25 μL with PBS buffer and incubated at 37°C for 40 min. Following this incubation, 250 μL of the red blood cell/phosphatidylcholine mixture was added, and the samples were incubated at 37°C for additional 15 min. Afterward, the samples were centrifuged at 3000 rpm for 10 min, and 200 μL of the supernatant was collected for absorbance measurement at 540 nm.

In the D. acutus venom control group, 20 μL of PBS-immunized serum was incubated with crude venom (10 μg), while the negative control group received an equivalent volume of physiological saline. The absorbance value obtained from the D. acutus group was considered to represent 100% indirect hemolytic activity. The relative hemolytic activity of each serum-treated group was calculated as follows:

$$\text{Relative hemolytic activity}\left(\text{\%}\right)\text{=}\frac{{{\text{OD}}_{\text{540 (serum group)}}}_{\;}}{{{\text{OD}}_{\text{540 (}\text{D. acutus}\text{group)}}}_{\;}}\text{×100\%}$$ (3)

### Neutralization of D. acutus venom-induced hemorrhage

The evaluation of hemorrhagic activity was performed based on the method described by previous studies [16,30,41]. The minimum hemorrhagic dose (MHD) is defined as the amount of venom required to induce a hemorrhagic lesion with a diameter of 10 mm following subcutaneous injection after 2 h. The MHD for D. acutus was determined to be 10 μg [35].

A total of 27 healthy female KM mice (weighing 17 ± 1.2 g) were randomly assigned to nine groups. Each group received a mixture of 100 μL of anti-ProMPLA serum with venom doses corresponding to the MHD, 2×MHD, and 4×MHD. These mixtures were incubated at 37°C for 40 min before subcutaneous injection into the dorsal skin of the mice. The anti-p2AMPLA serum was subjected to the same treatment protocol, while the D. acutus group received an equivalent volume of PBS-immunized serum. 

After 2 h of subcutaneous injection, the mice were euthanized via cervical dislocation. The dorsal skin was then carefully excised, and the diameter of the hemorrhagic lesions was measured to calculate the hemorrhagic area. Results were expressed as percentages, with the hemorrhagic area induced by the injection of MHD, 2×MHD, and 4×MHD doses of venom designated as 100% hemorrhagic activity.

### Neutralization of D. acutus venom-induced coagulation disruption

A total of twenty healthy female KM mice (weighing 17 ± 1.0 g) were randomly divided into four groups. In the normal control group, mice were administered 100 μL of PBS buffer via intraperitoneal injection. The sera-treated groups (anti-ProMPLA and anti-p2AMPLA) were treated with a mixture of 50 μL serum and an equal volume of D. acutus crude venom, administered at a dose corresponding to 1/2 × LD_50_ (where LD_50_ = 2.93 mg/kg [35]). In the D. acutus venom control group, 50 μL of PBS-immunized serum was incubated with an equal amount of snake venom. The mixture was incubated at 37°C for 40 min before intraperitoneal administration.

Three hours post-injection, fresh blood was collected via ocular puncture and immediately mixed with 0.109 M sodium citrate solution in a 9:1 (v/v) ratio to prevent coagulation. The mixture was then centrifuged at 3000 rpm for 10 to 15 min to obtain the upper layer of platelet-poor plasma. Additionally, the normal control group received an intraperitoneal injection of 100 μL of physiological saline, followed by the collection of platelet-poor plasma after three hours.

The coagulation parameters, including thrombin time (TT), prothrombin time (PT), activated partial thromboplastin time (APTT), and fibrinogen content (FIB), were assessed in plasma samples from all groups. These measurements were performed using the BCS-600 semi-automatic coagulation analyzer (Wuhan King Diagnostic Technology Co., Ltd), adhering to the manufacturer's instructions provided in the coagulation assay kit. 

### Neutralization of D. acutus venom-induced edema

The inhibition effect of serum on the edema induced of D. acutus snake venom was assessed using the following the method described by Dingwoke et al. [42] and Sobrinho et al. [43], with minor modifications. A total of 12 healthy female KM mice (weighing 16 ± 1.4 g) were randomly divided into four groups. In the D. acutus venom control group, 20 μL of PBS-immunized serum was mixed with 2 μg of D. acutus venom and incubated at 37°C for 40 min. The mixture was then injected into the plantar surface of the right hind paw. The same treatment protocol was applied to the anti-ProMPLA serum and anti-p2AMPLA serum groups. The negative control group received an equivalent volume of PBS buffer without venom or antibodies.

Using calipers (Ningbo Juren Measuring & Tools, China), the thickness of the right hind paw was measured at the following time points: 0, 0.5, 1, 1.5, 2, 2.5, and 3 h post-injection. The swelling rate was calculated according to the formula:

$$\text{Swelling rate (\%) =}\frac{\text{Thickness at different time points - Thickness before injection}}{\text{Thickness before injection}}\text{×100\%}$$ (4)

### Neutralization of sera on 2×LD_50_ *of D. acutus venom*

The protective effects of serum against the venom of D. acutus were performed based on the method described by previous studies [35,44], with minor modifications. The evaluation of hemorrhagic activity was performed based on the method described by previous studies, with minor modifications.

A total of 60 healthy female KM mice (weighing 16 ± 1.3 g) were randomly allocated into six groups, with 10 mice per group. Three groups received 100 μL of anti-ProMPLA, anti-p2AMPLA, or PBS-immunized serum mixed with 2×LD_50_ of crude venom from D. acutus venom (LD_50_ = 2.93 mg/kg), while the remaining three groups received 200 μL of the same treatments. All mixtures were incubated at 37°C for 40 min before being administered via intraperitoneal injection. The survival rates of the mice in each group were monitored over a 24-hour period.

### Data analysis

Data are presented as mean ± standard deviation, derived from experiments conducted at least three times. Statistical significance was assessed using one-way and two-way ANOVA, with multiple comparisons performed using GraphPad Prism Software (version 9). A p-value of less than 0.05 was considered statistically significant.

## Results and Discussion

### Construction and analysis of antigenic epitopes

To identify and construct antigenic epitopes for D. acutus venom MPs and PLA_2_s, a total of 16 amino acid sequences were selected and obtained from the UniProt database. These sequences included 13 derived from PI to PIII-SVMP and 3 from PLA_2_s (Table 1). The Bepipred-2.0 online analysis tool, which utilizes a cross-validated random forest regression algorithm trained on crystallography-derived epitope data, was used to predict potential antigenic sites. Residues with scores exceeding the default threshold of 0.5 were identified as candidate antigenic epitopes [45], as illustrated in Additional file 1.

**Table 1.** List of metalloproteinase and phospholipase A_2_ sequences of D. acutus snake venom.

UniProt accession n^o^	Protein name	Sequence length (AA)
*P-I MPs*		
Q9IAY0	Snake venom metalloproteinase H3	400
Q9IAY1	Snake venom metalloproteinase H2	400
Q9IAY2	Snake venom metalloproteinase H5	404
Q9IAY3	Snake venom metalloproteinase H1	400
Q9IAY4	Snake venom metalloproteinase H4	357
Q2EI26	Snake venom metalloproteinase AaPA	413
Q9PW35	Snake venom metalloproteinase acutolysin-A	413
Q9PW36	Snake venom metalloproteinase acutolysin-C	417
Q9W7S2	Snake venom metalloproteinase aculysin-1	417
*P-II MPs*		
Q9IAX6	Zinc metalloproteinase/disintegrin	466
Q9PWJ0	Zinc metalloproteinase/disintegrin	479
*P-III MPs*		
Q1PS45	Zinc metalloproteinase-disintegrin-like agkihagin	608
Q9W6M5	Zinc metalloproteinase-disintegrin-like agkihagin	610
*PLA*_*2*_		
O57385	Basic phospholipase A_2_ homolog acutohemolysin	138
Q1ZY03	Basic phospholipase A_2_ DAV-N6	138
Q7SID6	Acidic phospholipase A_2_	123

**Figure 1.**
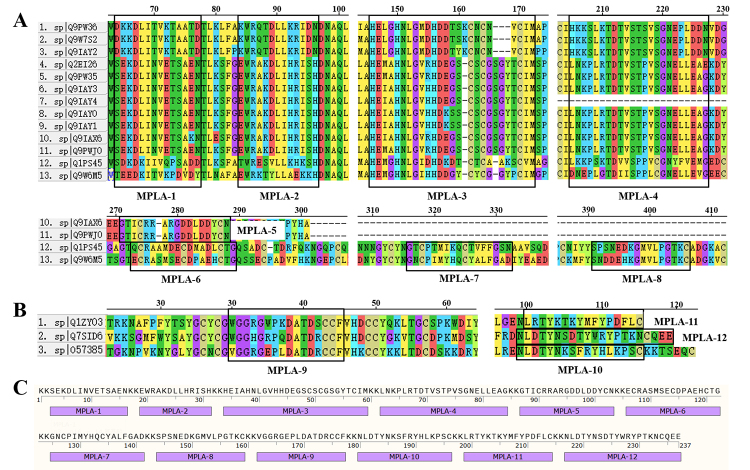
Antigenic epitope prediction and construction for D. acutus venom metalloproteinases (MPs) and phospholipases A_2_ (PLA_2_s). **(A, B)** Sequence alignment of 13 MPs and 3 PLA_2_s amino acid sequences, with the selected epitope regions outlined in black boxes. **(C)** The EpiMPLA amino acid sequence, showing the concatenated epitopes connected by KK linkers.

Sequence alignment using MEGA software revealed conserved homologous regions and functional motifs, which, combined with Bepipred predictions, guided the selection of 12 epitopes (MPLA-1 to MPLA-12) (Figure 1 A  and 1B). These epitopes were chosen based on their conservation, predicted antigenicity, and functional relevance. For example: MPLA-1 and MPLA-2 were selected based on their high sequence conservation and predicted antigenicity, which are critical for preserving the structural integrity of venom MPs. MPLA-3 includes the HEXXHNLXXHD and CIM motifs, which bind zinc ions and form the active site, leading to the degradation of extracellular matrix components and resulting in hemorrhage [46,47]. MPLA-4 was chosen due to its conserved nature, playing a role in stabilizing the metalloproteinase structure and enhancing substrate interaction. MPLA-5 features the well-known RGD motif, which inhibits platelet aggregation, thus contributing to the anticoagulant properties of the venom [48,49]. MPLA-6 contains the ECD motif, which can induce apoptosis, inhibit endothelial cell proliferation, and disrupt cell adhesion to extracellular matrix proteins, potentially impairing cell-matrix interactions [50,51]. MPLA-7 harbors the HQC motif, which interferes with the binding of integrin α1β1 to collagen and laminin, thereby impacting the coagulation process [52]. MPLA-8 was selected due to its high conservation and essential role in the structural integrity of PIII-type venom MPs [53]. MPLA-9 displays a high antigen index in PLA_2_, enhancing its ability to bind to B cell surface receptors, facilitating immune recognition. MPLA-10 to MPLA-12, located at the C-terminal region of the PLA_2_ sequences, are characterized by a series of positively charged and hydrophobic residues. These regions have been implicated in destabilizing the myofilament, causing plasma membrane disruption and excessive myofilament contraction, thereby contributing to the venom's myotoxic effects [10,54,55]. 

The selected epitopes were linked using a KK (Lys-Lys) linker to form a 237-amino acid peptide, EpiMPLA (Figure 1 C ). The KK linker was chosen for its dual functionality: (i) connecting protein regions to maintain the correct three-dimensional structure, and (ii) enabling cellular protease recognition and hydrolysis for independent antigen processing and presentation *in vivo* [41].

### Immunogens targeting EpiMPLA epitope from prokaryotic and eukaryotic expression systems

**Figure 2.**
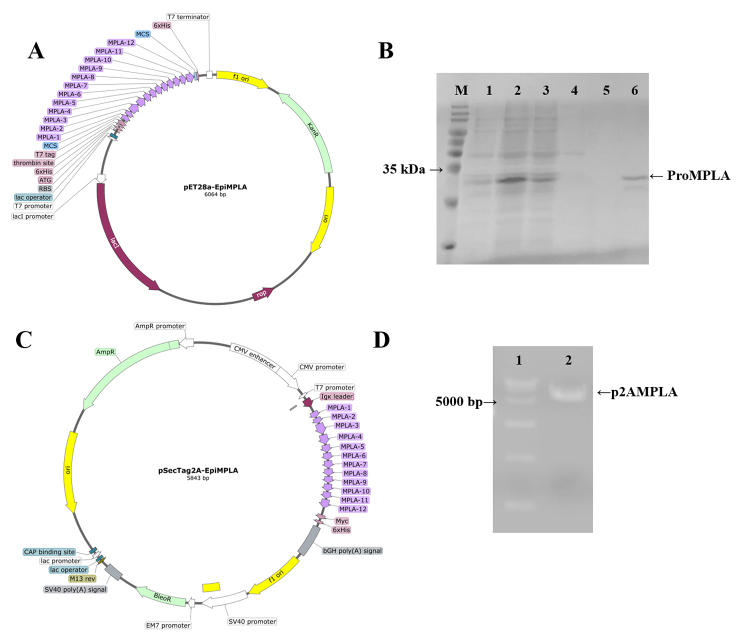
Results of immunogen ProMPLA and p2AMPLA preparation. **(A)** Map of the pET28a-EpiMPLA construct used for prokaryotic expression was derived using SnapGene 6.0 software (https://www.snapgene.com/). **(B)** Purification results of ProMPLA. Lane M: 15-120 kDa protein marker; lane 1: before IPTG induction; lane 2: after IPTG induction; lane 3: 5 mM imidazole wash; lane 4: 10 mM imidazole wash; lane 5: 20 mM imidazole wash; lane 6: 250 mM imidazole elution of ProMPLA (~30 kDa). **(C)** Map of the pSecTag2A-EpiMPLA construct used for eukaryotic expression was derived using SnapGene 6.0 software. **(D)** Identification of p2AMPLA plasmid following gel recovery. Lane 1: DL15000 DNA marker; lane 2: recombinant plasmid p2AMPLA.

To develop a prokaryotic expression system, the nucleotide sequence of EpiMPLA was optimized for E. coli codon usage. This optimized sequence was cloned into the BamH I and Hind III cleavage sites of the pET28a (+) prokaryotic expression vector (Figure 2 A ). The resulting recombinant protein, named ProMPLA, consists of 237 amino acids and includes an N-terminal His-tag with six histidine residues. Using the Expasy online tool (https://web.expasy.org/protparam/), the predicted molecular weight of ProMPLA was approximately 30.6 kDa.

Following transformation into DE3 cells, protein expression was induced with IPTG for 4 h. SDS-PAGE analysis revealed a distinct protein band within the 25-35 kDa range, consistent with the expected size of ProMPLA. Purification using Ni²⁺ affinity chromatography yielded a protein of approximately 30 kDa (Figure 2 B ), confirming the successful expression and purification of the recombinant protein.

For eukaryotic expression, the EpiMPLA sequence was codon-optimized for mice and cloned into the BamH I and Hind III sites of the pSecTag2A mammalian expression vector (Figure 2 C ). This vector is designed for efficient secretion of fusion proteins, featuring a CMV promoter for enhanced transcription and an Igκ-chain leader sequence (METDTLLLWVLLLWVPGSTGD) to facilitate precursor peptide activity [56,57]. The recombinant plasmid, p2AMPLA, was successfully constructed in DH5α cells and confirmed by agarose gel electrophoresis (Figure 2 D ). Sequence analysis verified that the p2AMPLA construct matched the intended design, ensuring its suitability for subsequent immunization studies.

### Antibody responses induced by immunization with recombinant proteins and gene plasmid

Following multiple immunizations with the recombinant protein ProMPLA and the recombinant plasmid EpiMPLA, fresh blood samples were collected from mice seven days after each immunization via orbital venipuncture. Serum was separated by centrifugation, and indirect ELISA was performed to assess the antibody titers against snake venom in the two groups. Initial ELISA results indicated low antibody levels after the first immunization, with a marked increase after the second and third immunizations.

**Figure 3.**
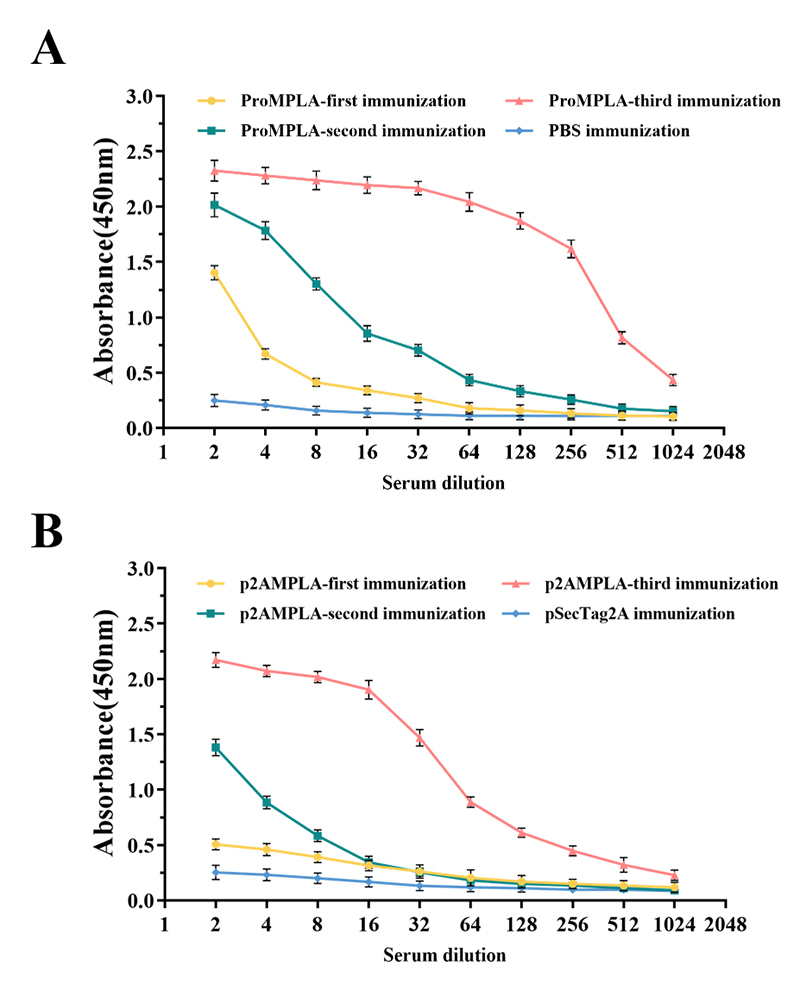
ELISA results of sera antibody detection against D. acutus venom. **(A)** Antibody titers of ProMPLA triple-immune serum against D. acutus venom. **(B)** Antibody titers of p2AMPLA triple-immune serum against D. acutus venom.

In the recombinant protein group, the anti-ProMPLA serum collected after the third immunization exhibited an A_450_ value of 0.43 at a 1:1024 dilution, compared to 0.11 for the PBS-immunized group (Figure 3 A ). This indicates a venom antibody titer exceeding 1:1024. In the recombinant plasmid group, the anti-p2AMPLA serum showed an A_450_ value of 0.32 at a 1:512 dilution, with the pSecTag2A-immune group yielding an A_450_ of 0.10 (Figure 3 B ), corresponding to a titer greater than 1:512.

These results demonstrate that both immunization strategies successfully induced antibodies capable of binding to D. acutus venom proteins. However, the recombinant protein-based immunization (anti-ProMPLA) elicited higher antibody titers compared to the gene-based approach (anti-p2AMPLA). This difference may be attributed to factors such as enhanced antigen presentation, stability of the recombinant protein, or more efficient immune recognition. Further investigation is needed to elucidate the underlying mechanisms, including the roles of antibody concentration, binding affinity, and neutralizing capacity. 

### Antibody specificity detection by Western blot

**Figure 4.**
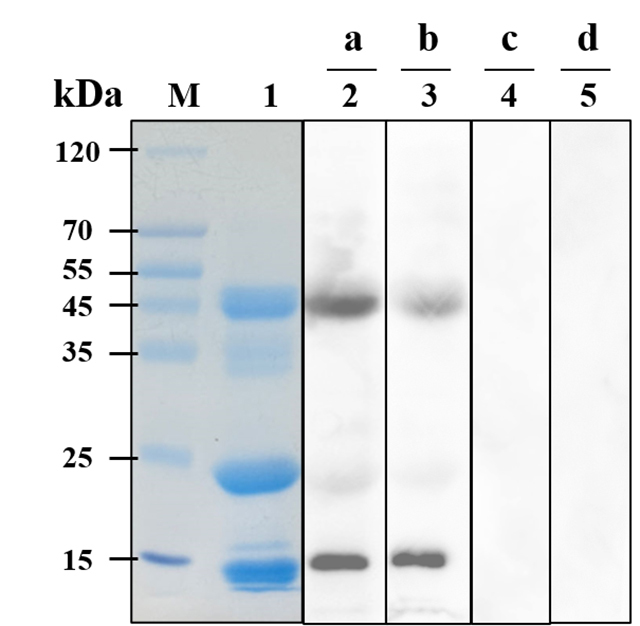
Western blot results of sera antibodies and D. acutus venom. Lane M: 15-120 kda protein marker; lanes 1-5: 30 μg of D. acutus venom; (a) anti-ProMPLA serum; (b) anti-p2AMPLA serum; (c) PBS-immunized serum; (d) pSecTag2A-immune serum.

To assess the specificity of antibodies generated in immunized mice for D. acutus venom metalloproteinases (MPs) and phospholipases A_2_ (PLA_2_s), Western blot (WB) analysis was performed. This method, widely used for detecting antigen-antibody interactions, confirmed that both anti-ProMPLA and anti-p2AMPLA sera specifically bound to venom components, as evidenced by distinct bands (Figure 4, lanes 2 and 3).

SDS-PAGE analysis of D. acutus venom revealed dominant protein bands in the 12-15 kDa, 20-25 kDa, and 45-50 kDa ranges (Figure 4, lane 1), which is consistent with previous studies [1,36,58,59]. In particular, PIII-SVMPs were identified in the 45-50 kDa range, while PI/PII-SVMPs appeared in the 20-25 kDa range. PLA_2_s, with a molecular weight around 14 kDa, were also detected, aligning with the findings of Xie et al. [58], who identified a 14.8 kDa PLA_2_ from D. acutus. 

The sera showed stronger reactivity toward PIII-SVMPs and PLA_2_s compared to PI/PII-SVMPs, as indicated by the intensity of the bands. In contrast, control sera from PBS-immunized and pSecTag2A-immunized groups showed no significant binding (Figure 4, lanes 4 and 5), confirming the specificity of the interactions observed with anti-ProMPLA and anti-p2AMPLA sera. These results demonstrate that immunization with ProMPLA and p2AMPLA efficiently induces antibodies targeting MPs and PLA_2_s in D. acutus venom, providing critical insights into the mechanisms of antibody-mediated venom neutralization.

### Inhibition of D. acutus venom metalloproteinase activity by anti-ProMPLA and anti-p2AMPLA sera

**Figure 5.**
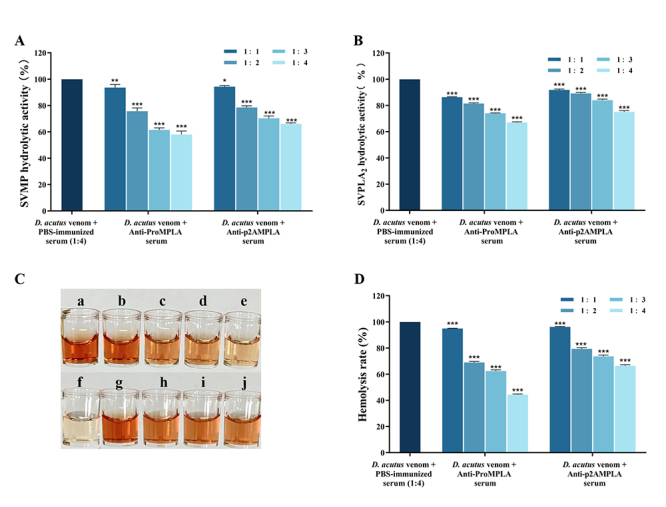
Inhibition of D. acutus venom enzyme activity by anti-ProMPLA and anti-p2AMPLA sera. **(A)** Hydrolytic activity of D. acutus SVMP (%). **(B)** Hydrolytic activity of D. acutus SVPLA_2_ (%). **(C)** Representative image of (a) 10 μg D. acutus with 20 μL PBS-immunized serum; (b-e) 10 μg D. acutus with anti-ProMPLA at ratios of 1:1, 1:2, 1:3, and 1:4 (v/v); (f) 25 μL physiological saline; (g-j) 10 μg D. acutus with anti-p2AMPLA at ratios of 1:1, 1:2, 1:3, and 1:4 (v/v). **(D)** Hemorrhage rate (%) of indirect hemolysis induced by D. acutus snake venom. ***Significant differences compared to the PBS-immunized serum group; p < 0.001.

The experiment assessed the inhibitory effects of anti-ProMPLA and anti-p2AMPLA sera on MPs activity by comparing absorbance changes across different serum volumes (Figure 5 A ). Compared to the D. acutus venom control group, both sera inhibited the hydrolysis activity of SVMPs. Specifically, when venom was mixed with serum at ratios of 1:1, 1:2, 1:3, and 1:4 (v/v), anti-ProMPLA serum reduced enzyme activity by 7%, 24%, 39%, and 41%, respectively. Similarly, anti-p2AMPLA serum reduced activity by 6%, 21%, 30%, and 34%, respectively, under the same conditions.

These results demonstrate that 20 μL of anti-ProMPLA serum effectively inhibited the metalloproteinase activity by 41% when tested against 5 μL of crude venom (10 μg). Although the two sera showed partial recognition of the toxin, the epitope design strategy successfully guided the preparation of antibody sera with neutralizing activity against metalloproteinases.

### *Inhibition of D. acutus venom phospholipase A*_2_ activity by anti-ProMPLA and anti-p2AMPLA sera

PLA_2_ hydrolytic activity in D. acutus snake venom has been extensively documented. PLA_2_ acts on lecithin in egg yolk mixed with agar, leading to the formation of clear zones in the agar, which are used to quantify and assess PLA_2_ activity [60,61]. Compared to the 100% SVPLA_2_ hydrolytic activity observed in the D. acutus venom control group, both anti-ProMPLA and anti-p2AMPLA sera exhibited significant inhibitory effects. Specifically, when venom was mixed with serum at ratios of 1:1, 1:2, 1:3, and 1:4 (v/v), anti-ProMPLA serum reduced enzyme activity by 14%, 18%, 26%, and 33%, respectively. Similarly, anti-p2AMPLA serum reduced activity by 8%, 11%, 16%, and 25% under the same conditions (Figure 5 B ).

These results demonstrate that when 20 μL of serum was applied to 5 μL of crude venom (10 μg), the inhibition rates reached 33% for anti-ProMPLA and 25% for anti-p2AMPLA. Although the sera partially recognized the toxin, a significant portion of the toxin retained SVPLA_2_ hydrolytic activity.

### Inhibition of D. acutus venom-induced hemolysis by anti-ProMPLA and anti-p2AMPLA sera

In the presence of Ca^2+^ ions, co-incubation of snake venom with red blood cells and phosphatidylcholine for 15 min led to significant hemolysis, as evidenced by the red coloration of the supernatant compared to the negative control group (Figure 5 C-a  and 5C-f). However, with increasing serum concentration, the extent of hemolysis decreased. When venom was incubated with serum at ratios of 1:1, 1:2, 1:3, and 1:4 (v/v), anti-ProMPLA serum reduced the hemolysis rate by 5%, 31%, 38%, and 56%, respectively. Similarly, anti-p2AMPLA serum reduced the activity by 4%, 21%, 26%, and 34% (Figure 5 D ).

These results demonstrate that 20 μL of anti-ProMPLA serum could reduce the hemolysis rate of 5 μL of crude venom (10 μg) by a maximum of 56%. During the incubation phase, the serum successfully recognized some of the toxin, thereby preventing complete rupture of the blood cells.

### Neutralization of D. acutus venom-induced hemorrhage by anti-ProMPLA and anti-p2AMPLA sera

**Figure 6.**
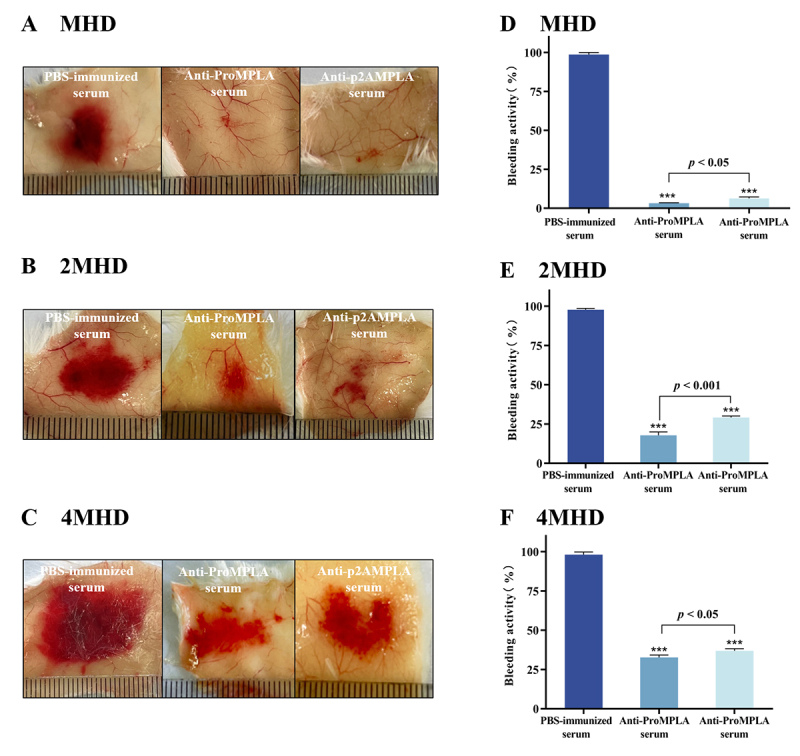
Neutralization of D. acutus venom-induced hemorrhage by anti-ProMPLA and anti-p2AMPLA sera. Representative images of subcutaneous hemorrhagic areas induced by D. acutus venom at doses of **(A)** 1×MHD, **(B)** 2×MHD, and **(C)** 4×MHD, incubated with PBS-immunized, anti-ProMPLA, or anti-p2AMPLA sera. Hemorrhagic activity corresponding to D. acutus venom at doses of **(D)** 1×MHD, **(E)** 2×MHD, and **(F)** 4×MHD, incubated with PBS-immunized, anti-ProMPLA, or anti-p2AMPLA sera. The values represent the mean percentage of hemorrhagic area relative to the PBS-immunized serum group, which is set at 100%. ***Significant differences compared to the PBS-immunized serum group; p < 0.001.

A key characteristic of D. acutus envenomation is hemorrhage. To evaluate the neutralizing efficacy of two sera, a subcutaneous hemorrhage neutralization assay was performed, where the venom-induced hemorrhagic area was defined as 100% hemorrhagic activity. At the MHD, both anti-ProMPLA and anti-p2AMPLA serum effectively recognized venom-induced hemorrhage (p < 0.001), and compared with the D. acutus venom control group, both sera near-complete inhibition of the hemorrhagic effect (p < 0.05) (Figure 6 A and 6D). At 2×MHD, both sera significantly reduced the hemorrhagic area compared to the D. acutus venom control group (p < 0.001), with the color of the hemorrhagic spots becoming noticeably lighter (Figure 6 B ). Anti-ProMPLA reduced the hemorrhagic area by 82.2%, outperforming anti-p2AMPLA, which achieved a 70.8% reduction (p < 0.001) (Figure 6 E ).

At 4×MHD, the diameter of the subcutaneous hemorrhagic spots in the D. acutus venom control group reached up to 25 mm. However, both sera continued to significantly reduce the hemorrhagic area (p < 0.001) (Figure 6 C ). Anti-ProMPLA reduced the area by 67.3%, while anti-p2AMPLA achieved a 63.1% reduction (p < 0.05) (Figure 6 F ). These results demonstrate that both sera can mitigate subcutaneous hemorrhage caused by D. acutus venom, with anti-ProMPLA exhibiting superior neutralizing efficacy compared to anti-p2AMPLA.

### Neutralization of D. acutus venom-induced coagulation disruption by anti-ProMPLA and anti-p2AMPLA sera

*D. acutus* venom, known for its hemorrhagic effects, contains metalloproteinases that disrupt blood coagulation by hydrolyzing coagulation factors, fibrinogen, and collagen. In mice injected with a mixture of D. acutus venom and PBS-immunized serum (control group), significant coagulation abnormalities were observed, with thrombin time (TT) prolonged to 78.50 s, prothrombin time (PT) to 36.74 s, and activated partial thromboplastin time (APTT) to 46.91 s. Fibrinogen (FIB) levels were reduced to 0.65 g/L (Table 2, p < 0.01), indicating impaired coagulation and resulting hypofibrinogenemia and uncontrolled bleeding.

**Table 2.** Effects of anti-ProMPLA and anti-p2AMPLA sera on coagulation function indices induced by D. acutus venom in mice

	TT (s)	PT (s)	APTT (s)	FIB (g/L)
Normal	17.25 ± 1.32	9.00 ± 0.23	17.44 ± 0.46	2.59 ± 0.06
1/2 ×LD_50_ + PBS-immunized serum	78.50 ± 1.73^^^	36.74 ± 2.62^^^	46.91 ± 1.74^^^	0.65 ± 0.10^^^
1/2 ×LD_50_ + Anti-ProMPLA serum	36.66 ± 1.72^^#^	13.00 ± 0.99^^#^	20.73 ± 0.71^^#^	1.71 ± 0.10^^#^
1/2 ×LD_50_ + Anti-p2AMPLA serum	20.88 ± 0.62^^#^	13.33 ± 0.69^^#^	33.90 ± 1.25^^#^	1.32 ± 0.14^^#^

^^^p < 0.01 (compared to the normal group); ^#^p < 0.001 (compared to 1/2 × LD_50_ + PBS-immunized group).

Incubation of venom with anti-ProMPLA and anti-p2AMPLA sera led to significant improvements in coagulation parameters. TT decreased to 36.66 s and 20.88 s, PT shortened by 13.00 s and 13.33 s, APTT reduced to 20.73 s and 33.90 s, and FIB levels were partially restored to 1.71 g/L and 1.32 g/L, respectively. While these values remained lower than the normal control group, they were significantly improved compared to the venom control group (p < 0.001). These findings demonstrate that sera neutralization mitigates venom-induced coagulopathy, highlighting the potential of epitope-based immunization strategies in counteracting snake envenomation.

### Neutralization of D. acutus venom-induced edema by anti-ProMPLA and anti-p2AMPLA sera

Edema induced by D. acutus venom is primarily caused by components such as PLA_2_s and MPs. PLA_2_ hydrolyzes phospholipids in cell membranes, leading to cellular content leakage and triggering localized inflammatory responses. This process activates inflammatory signaling pathways, resulting in the release of mediators like IL-1, IL-6, and TNF-α. These mediators increase vascular permeability, causing fluid leakage into interstitial tissues and leading to edema [60,62,63]. Similarly, MPs degrade collagen and laminin in the extracellular matrix and vascular walls, further exacerbating vascular permeability and fluid leakage.

**Figure 7.**
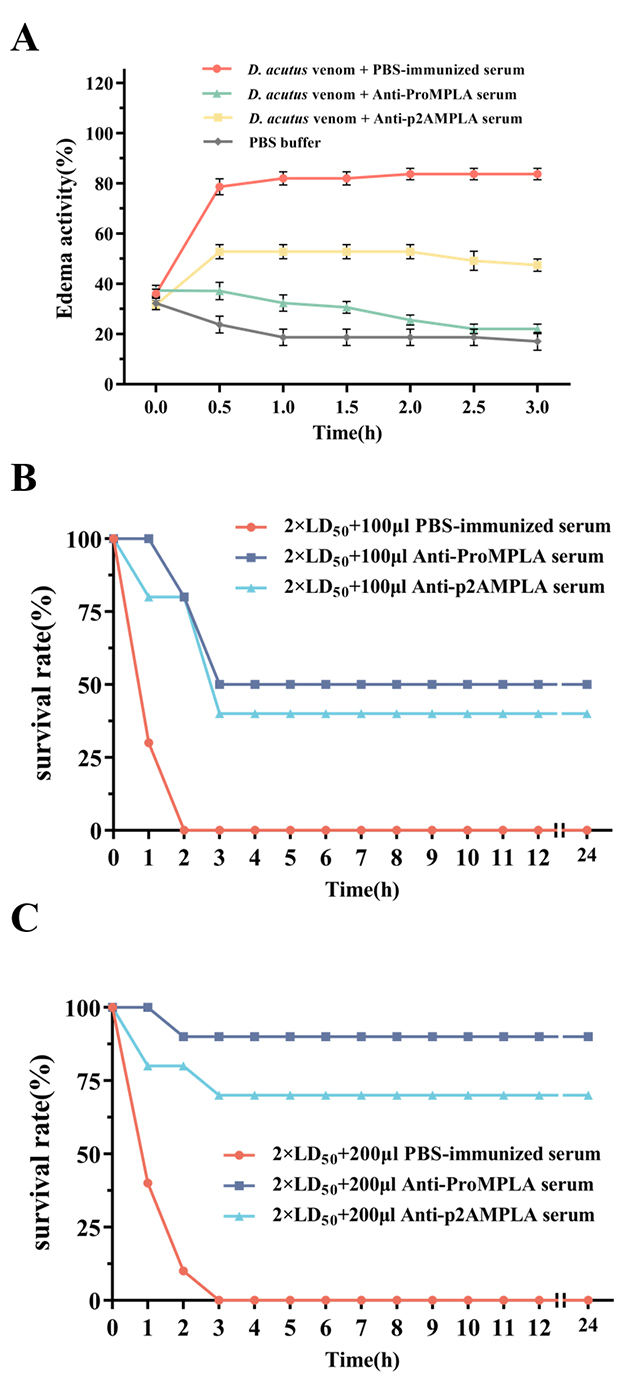
Effect of anti-ProMPLA and anti-p2AMPLA sera on toxic responses induced by D. acutus venom in mice. **(A)** Assessment of edema activity. After preincubation of 2 μg of D. acutus venom with PBS-immunized, anti-ProMPLA, or anti-p2AMPLA sera, the venom was injected into the mice's hind paws. The thickness of the paws was measured at 0, 0.5, 1, 1.5, 2, 2.5, and 3 h post-injection. Edema activity was calculated relative to the thickness at 0 h. PBS buffer alone served as the negative control group. Neutralizing effect of anti-ProMPLA and anti-p2AMPLA sera on 2×LD_50_ of D. acutus venom. The results are presented as the survival rate of mice after 2×LD_50_ D. acutus venom within 24 h after **(B)** 100 μL or **(C)** 200 μL PBS-immunized, anti-ProMPLA, and anti-p2AMPLA sera preincubation. Values represent the survival rate of the mice compared to baseline measurements prior to injection.

In the edema inhibition assay, venom injection led to a peak swelling rate of 85.3% at 0.5 h, with continued worsening of edema over time. However, when venom was pre-incubated with anti-ProMPLA serum, the swelling rate of the mouse paw decreased significantly, reaching 22.0% at 3 h, comparable to the negative control group (17.0%) (Figure 7 A ). In contrast, venom incubated with anti-p2AMPLA serum exhibited an initial peak swelling rate of 54.5% at 0.5 h, which decreased to 47.5% at 3 h.

###  Neutralizing and protective effects of anti-ProMPLA and anti-p2AMPLA sera against *D. acutus* venom 

To assess the neutralizing capability of the sera, a toxicity protection assay was conducted. At a dose of 2×LD_50_, all mice succumbed to venom toxicity. However, pre-treatment with 100 μL of anti-ProMPLA and anti-p2AMPLA sera increased survival rates to 50% and 40%, respectively (Figure 7 B ). When the serum dose was raised to 200 μL, survival rates further improved to 80% and 60% (Figure 7 C ), demonstrating the sera's substantial protective effects against D. acutus venom toxicity.

Systemic hemorrhage and mortality are critical consequences of snakebites. Previous studies have explored epitope-based antivenom strategies. For example, Gutiérrez [64] used venomics and antivenomics to identify linear epitopes in snake venom toxins, producing sera with high specificity. Similarly, Laustsen et al. [65] employed computer-assisted design to target neurotoxic epitopes in black mamba venom (Dendroaspis polylepis), significantly improving survival rates in envenomated mice. These studies underscore the potential of epitope-based approaches in antivenom development.

In this study, both anti-ProMPLA and anti-p2AMPLA sera demonstrated efficacy against D. acutus venom-induced toxicity, with anti-ProMPLA showing superior performance. This difference may be attributed to several factors: (1) inefficient uptake of DNA immunogens by host cells, (2) suboptimal conversion of DNA into antigenic proteins in vivo, (3) susceptibility of naked DNA to nuclease degradation, and (4) insufficient immune response enhancement by vectors and adjuvants. These limitations likely contribute to the slightly lower efficacy of gene-based immunization compared to recombinant protein-based approaches, which remain more suitable for antivenom research.

### Limitations

Although the potential of epitope-based immunization for antivenom development is demonstrated in this study, several limitations should be acknowledged. First, some of the identified epitopes may share sequence homology with human proteins, raising the possibility of anti-human antibody responses. To address this, epitope selection will be refined using comprehensive BLAST analysis, with a focus on prioritizing epitopes unique to venom toxins. However, further experimental validation will be required to confirm the specificity and safety of the generated antibodies. Second, the current study focused on a limited number of venom components (MP and PLA_2_), and the efficacy of the sera against other venom toxins has yet to be evaluated. Third, it remains to be tested whether the epitopes in the designed immunogens are functionally presented. Future studies will explore alternative linkers (e.g., GG linkers) and modified epitope compositions to optimize epitope presentation and assess the functional activity of the antisera. Finally, although promising in vivo results were obtained, the translation of these findings to clinical applications will require further optimization and evaluation in more complex models.

## Conclusion

This study used bioinformatics tools to identify 12 antigenic epitopes from D. acutus venom MPs and PLA_2_s, leading to the design of the composite antigen peptide EpiMPLA. Recombinant immunogens (ProMPLA and p2AMPLA) were expressed and used to immunize mice. The immune response generated strong antibodies that effectively inhibited MP and PLA_2_ enzymatic activities. In vivo, immunized mice showed significant protection against venom-induced bleeding and edema. Recombinant protein-based immunization, which presented multiple epitopes in a stable form, resulted in stronger ability to alleviate snake venom effects compared to gene-based immunization. These findings highlight the advantages of recombinant protein strategies for optimizing antivenom development. Future research will focus on refining epitope selection, enhancing antibody specificity, and evaluating efficacy in more complex envenomation models to develop safer, more effective therapies for snakebite.

### Abbreviations

APTT: activated partial thromboplastin time; CLP: C-type lectin proteins; ECD: Glu-Cys-Asp; FCA: Freund's Complete Adjuvant; FIA: Freund's Incomplete Adjuvant; FIB: fibrinogen content; HQC: His-Gin-Cys; HRP: horseradish peroxidase; IEDB: Immune Epitope Database; IPTG: isopropyl-beta-D-thiogalactopyranoside; LD_50_: median lethal dose; MHD: minimum hemorrhagic dose; MP: metalloproteinase; PCV2: porcine circovirus type 2; PLA_2_: phospholipase A_2_; PT: prothrombin time; RGD: Arg-Gly-Asp; TMB: 3, 3’,5 ,5’-tetramethylbenzidine; TNF-α: tumor necrosis factor-α; TT: thrombin time.

## Data Availability

All data generated or analyzed during this study are included in this article and its additional files.

## Supplementary material

Additional file 1. Antigenic epitopes of **(A)** metalloproteinase and **(B)** phospholipase A_2_ from Deinagkistrodon acutus venom predicted by IEDB-Bepipred 2.0 online server, with a threshold of 0.5. The yellow sections represent the predicted antigenic epitope regions.
