# Supplementary material for: Epitope-based antibody development against metalloproteinases and phospholipases A2 from Deinagkistrodon acutus venom
Source: J Venom Anim Toxins Incl Trop Dis. 2025 May 9;31:e20240060. doi: 10.1590/1678-9199-JVATITD-2024-0060 (PMC12063738; doi:10.1590/1678-9199-JVATITD-2024-0060)
Supplement: Additional file 1. [file 1678-9199-jvatitd-31-e20240060-s1.pdf]

J Venom Anim Toxins incl Trop Dis
https://doi.org/10.1590/1678-9199-JVATITD-2024-0060

Supplementary Material to: "Epitope-based antibody development against metalloproteinases and phospholipases A_2 from *Deinagkistrodon acutus* venom"

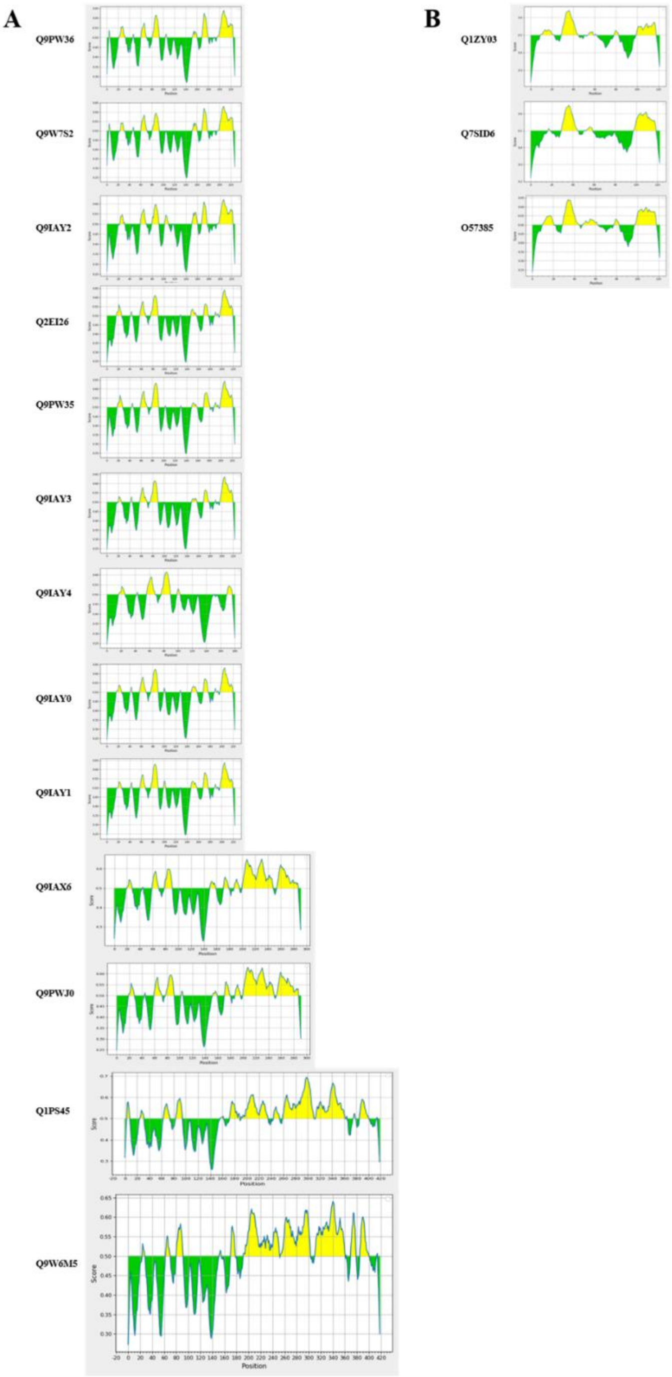

Additional file 1. Antigenic epitopes of **(A)** metalloproteinase and **(B)** phospholipase A_2 from *Deinagkistrodon acutus* venom predicted by IEDB-Bepipred 2.0 online server, with a threshold of 0.5. The yellow sections represent the predicted antigenic epitope regions.
